# Targeted identification of genomic regions using TAGdb

**DOI:** 10.1186/1746-4811-6-19

**Published:** 2010-08-20

**Authors:** Daniel J Marshall, Alice Hayward, Dominic Eales, Michael Imelfort, Jiri Stiller, Paul J Berkman, Terry Clark, Megan McKenzie, Kaitao Lai, Chris Duran, Jacqueline Batley, David Edwards

**Affiliations:** 1 University of Queensland, Australian Centre for Plant Functional Genomics, School of Land, Crop and Food Sciences, Brisbane, QLD 4067, Australia; 2 University of Queensland, ARC Centre of Excellence for Integrative Legume Research and School of Land, Crop and Food Sciences, Brisbane, QLD 4067, Australia

## Abstract

**Background:**

The introduction of second generation sequencing technology has enabled the cost effective sequencing of genomes and the identification of large numbers of genes and gene promoters. However, the assembly of DNA sequences to create a representation of the complete genome sequence remains costly, especially for the larger and more complex plant genomes.

**Results:**

We have developed an online database, TAGdb, that enables researchers to identify paired read sequences that share identity with a submitted query sequence. These tags can be used to design oligonucleotide primers for the PCR amplification of the region in the target genome.

**Conclusions:**

The ability to produce large numbers of paired read genome tags using second generation sequencing provides a cost effective method for the identification of genes and promoters in large, complex or orphan species without the need for whole genome assembly.

## Background

The availability of a reference genome sequence is a goal of many researchers in crop genomics. The first plant genomes to be sequenced were Arabidopsis and rice [1,2], applying standard Sanger sequencing of tiled genomic fragments maintained in bacterial artificial chromosome (BAC) vectors. Plant genome projects are rapidly changing pace with the application of new technology and the genomes of several plant species have now been determined [3] with researchers quickly adopting second generation sequencing to gain insight into their favourite genome. Many crop genomes are large, complex and often polyploid, making genome sequencing a major challenge [4,5]. Without a complete genome sequence, researchers are often limited to the available sets of expressed sequence tags (ESTs) or genome survey sequences.

The advent of second generation sequencing enables the production of large quantities of genome sequence data at relatively low cost. Second generation sequence data takes the form of vast numbers of relatively short sequence reads, often produced as pairs with a known orientation and approximate distance between the pair. While the assembly of this data to produce a representation of the genome requires highly redundant sequencing and a large number of overlapping sequence reads, only relatively low coverage is required for the identification of genes and gene promoters. However, there is a challenge to store, interrogate and visualise the quantity of sequence tag data required for such analysis [6]. We have developed TAGdb, a web based tool for the identification of Illumina GAII paired read sequences that match a query sequence. When combined with PCR amplification and sequencing, it is possible to determine the sequence of specific local genomic regions. This tool is applicable for gene and promoter discovery in a wide range of species and greatly facilitates comparative genomics and molecular marker discovery in orphan crops or those with large and complex genomes.

## Construction and content

TAGdb is a web-based query tool for aligning query sequences to an existing database of paired short read data. The system has been developed using Perl and MySQL and runs on a public web server (http://flora.acpfg.com.au/tagdb/). The interface allows researchers to upload or input a FASTA formatted nucleotide sequence up to 5000 base pairs long for comparison with one or more paired read sequence libraries. The input sequence is aligned with short reads of significant identity using MEGABLAST [7], and the results visualised using a custom web interface. Each submitted job has a unique identifier, and an email is sent to the user once the job has completed. The processing time per search varies depending on the length of the input sequence and number of matching reads, but generally, searches are completed and results are returned within 20 seconds to 5 minutes. The database currently hosts data for *Brassica*, wheat, barley, *Pongamia pinnata *and *Nicotiana alata*, and details of these data sets are available on the help pages. Additional Illumina paired short read sequence data may be hosted on request.

## Utility

### TAGdb output for the *AtWD40 *genomic region

**Figure 1:**
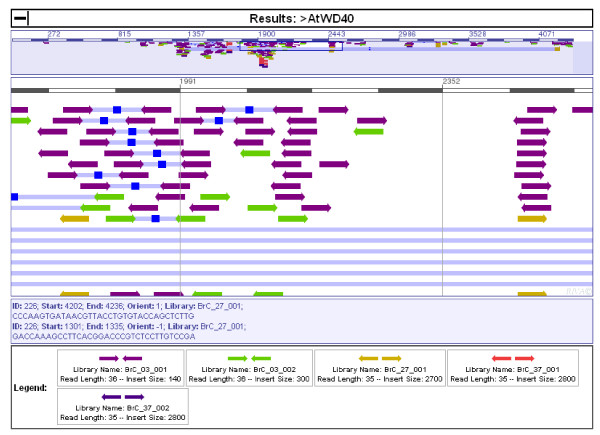
**TAGdb**. Screenshot of TAGdb showing the alignment of short reads from *Brassica rapa *cv Chiifu with the *AtWD40 *query sequence.

The Arabidopsis genomic region containing AT3G51930 (*AtWD40*) was selected to demonstrate the use of TAGdb for obtaining orthologous genomic sequences in *B. rapa*. More than 370 *Brassica rapa *tags aligned to this Arabidopsis reference, the majority of which align within the *AtWD40 *coding region (Figure 1), demonstrating the high level of sequence conservation within the gene. Fewer tags were identified from *B. oleracea *and *B. nigra *reflecting the lower overall tag coverage for these species. No tags were identified within wheat, barley or *Pongamia *libraries (Table 1).

**Table 1:** The number of individual and paired reads from different species matching the *AtWD40 *genomic region.

Dataset	Read pairs	Single reads
*B. rapa*	45	332

*B. oleracea*	12	68

*B. nigra*	14	88

Barley	0	0

Wheat	0	0

*Pongamia*	0	0

**Total**	**71**	**488**

### Sequencing the *B. rapa WD40 (BrWD40) *genomic region

**Figure 2:**
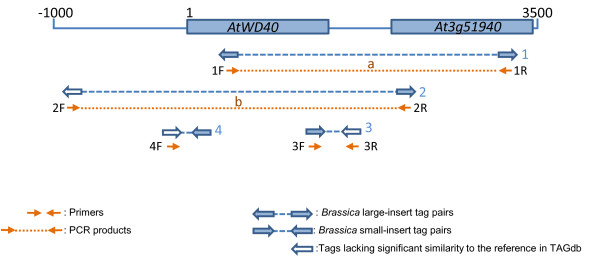
**Positions of the *B. rapa *tags and PCR primer sequences with respect to the *AtWD40 *genomic region**. Tag pairs and primer pairs used for PCR are labelled as per Table 2.

**Figure 3:**
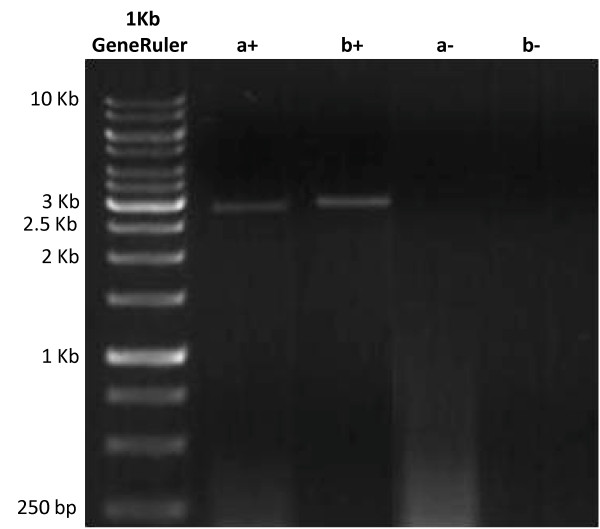
**Amplification of *BrWD40***. 1% TAE-agarose gel showing amplification products (+) and negative controls (-) for PCR products (a) and (b).

PCR primer pairs were designed to amplify the *BrWD40 *genomic region (Figure 2). Two primer pairs 1F and 1R, and 2F and 2R, were designed to generate overlapping 3 Kbp products covering the *BrWD40 *coding region as well as approximately 1 Kbp upstream and 2 Kbp downstream of the coding sequence (Figure 2 and Figure 3). As shown, primer 2F was designed from a *B. rapa *tag that was predicted to reside approximately 1000 bp 5' of the *WD40 *gene based on the location of its tag pair, as there were few *B. rapa *tags matching 5' of the *AtWD40 *gene.

**Figure 4:**
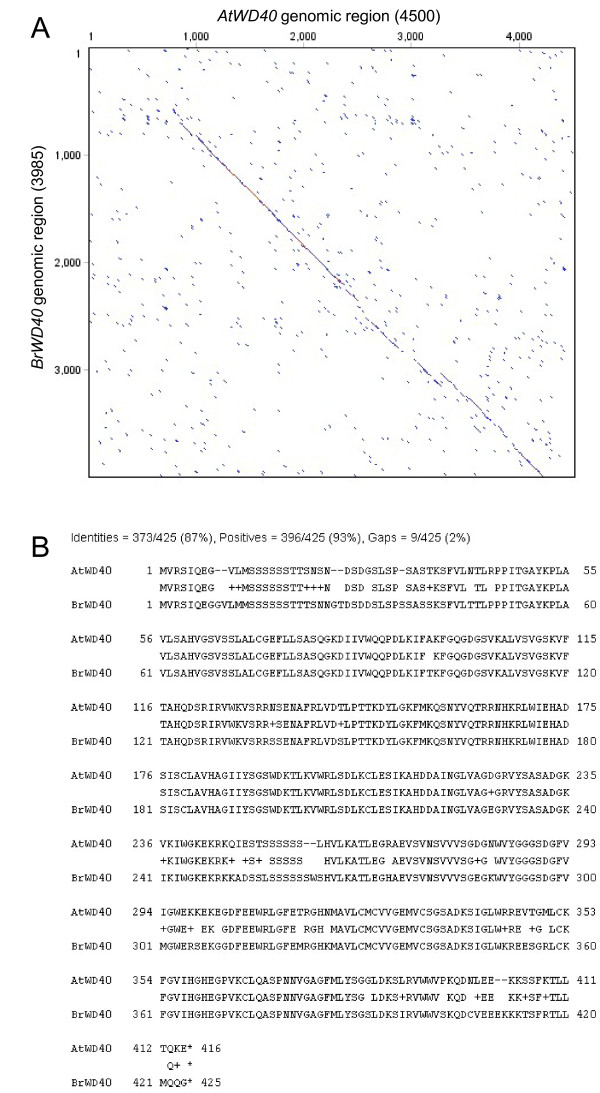
**Comparison of the AtWD40 and BrWD40 genomic regions**. (A) Dot plot comparison of the *AtWD40 *and *BrWD40 *genomic regions. (B) Alignment of the predicted amino acid sequences for *AtWD40 *and *BrWD40*.

A dotplot of the alignment of the *AtWD40 *reference region with the *B. rapa *cv. Chiifu genomic consensus sequence derived from products (a) and (b) is shown in Figure 4A. The *B. rapa *genomic consensus shares 65% overall nucleotide identity with the corresponding region in Arabidopsis. Of this, 86% sequence identity exists between the *AtWD40 *and predicted *BrWD40 *coding regions, 60% similarity exists between the *AtWD40 *and *BrWD40 *downstream regions (exclusive of the adjacent AT3G51940 downstream coding fragment) and 41% identity exists between the *WD40 *upstream regions. An alignment between the *AtWD40 *and putative *BrWD40 *protein sequences revealed 87% identity (Figure 4B) and a comparison of *BrWD40 *with the Arabidopsis genome using BLAST [8] returned the single *AtWD40 *reference sequence.

A large number of tags matching the *AtWD40 *region were identified from *B. rapa *with fewer tags identified for *B. oleracea *and *B. nigra*, reflecting the relative abundance of reads for these species in the dataset. No tags were identified for *Pongamia*, wheat or barley. Given the size of the wheat genome and the relatively low sequence coverage of this genome in our dataset, it is not possible to conclude whether an ortholog of AtWD40 is absent in wheat. The higher sequence coverage for wheat chromosome arm 7DS, barley and *Pongamia*, and the number of orthologous tags identified when these datasets are searched with rice and soybean genes respectively (data not shown), suggests that there is no orthologous gene with sequence identity to *AtWD40 *in *Pongamia*, barley, or on wheat chromosome arm 7DS.

Assembly of the *B. rapa *tags with the *AtWD40 *genomic sequence demonstrated that the majority of reads aligned with the coding region (Figure 1). This reflects the greater conservation of sequence due to evolutionary constraints within coding regions. PCR amplification using primers specific to tags 5', 3' and within the coding region of *BrWD40*, amplified single products (Figure 3). Sequencing these products demonstrated that they corresponded to the *B. rapa *region orthologous to *AtWD40 *(Figure 4).

## Conclusions

We have developed an online system for the identification and visualisation of second generation paired sequence tags matching to query sequences. While relatively simple in its concept, the system provides a powerful means to interrogate the vast quantity of data produced by the latest sequencing technologies in a user-friendly and intuitive manner, enabling the identification and cloning of novel genes and the surrounding genomic regions.

We have demonstrated the application of TAGdb for gene and promoter discovery in genomes where complete genome sequences are unavailable. We highlight the ability to amplify and sequence less conserved genomic regions, such as promoter sequences, using paired sequence tags where only one tag may align significantly to a query sequence. This tool can be applied for any species where paired read sequence data is available. While the current datasets are limited to a few species, the generation of short paired read sequence data is becoming increasingly common and this approach is likely to become a standard method for the discovery of genes, promoters and genetic variation in a wide range of species. While the current tool is specifically designed for Illumina paired reads, similar data produced by other sequencing platforms may also be hosted.

## Availability and requirements

TAGdb is freely available at http://flora.acpfg.com.au/tagdb/.

## Methods

### Identification of Brassica WD40 orthologs

A 4.5 Kbp fragment of the Arabidopsis genomic region beginning 1000 bp upstream of the transducin/WD-40 repeat family gene AT3G51930 (referred to as *AtWD40*) was used to query all currently available TAGdb datasets (Table 1). PCR primer pairs were designed from *Brassica rapa *tags aligning to the query sequence for the amplification of the *WD40 *genomic region in *Brassica rapa *cv. Chiifu (Table 2). PCR products (a) and (b) were amplified from 60 ng of *Brassica rapa *cv. Chiifu DNA using 0.5 μM forward and reverse oligonucleotide primer, 1 U of Phusion Hot Start High-Fidelity DNA Polymerase (Finnzymes), 1× Phusion GC Buffer (Finnzymes) with 1.5 mM MgCl_2_, and 200 μM each dNTP in a PTC-200 Thermocycler (MJ Research). Cycling conditions were 98°C for 30 sec followed by 35 cycles of 98°C for 10 s, 63°C for 30 s and 72°C for 1 min, with a final extension of 72°C for 10 min. Amplified products were visualised under UV light on 1% TAE-agarose gels containing Ethidium Bromide and using the GeneRuler™ 1 Kb marker as a size standard. PCR products (a) and (b) were cloned using the pGEM^®^-T-easy (Promega) and pCR^®^-XL-Topo^® ^(Invitrogen) vector systems respectively and sequenced using T7 and SP6 or M13R primers, and the internal primers 1F, 2R, 3F, 3R, 4F (Table 2) and WD40_3pseqR (5'-TGGAAGAGATTAGGTGAAATGTGA-3'). A consensus sequence for the *BrWD40 *region (3985 bp) was generated in Geneious Pro [9] from a contig assembly of sequenced products. Alignments and dotplots (window size 10, threshold 35) were generated in Geneious Pro using MUSCLE [10] and ClustalW [11] with default settings.

**Table 2:** PCR primer sequences and tags used for the amplification and sequencing of the *BrWD40 *genomic region.

Primer pair	Primer sequence (5'-3')	Tag name	Primer position relative to *AtWD40 *start codon (bp)	*B. rapa *product size (bp)
1F	TCGGACAAGGAGACGGGTCC	BrC_27_001_56-123-1844_2	290	a: 2862
1R	AAGAGCTGGTACACAGGTAACG	BrC_27_001_56-123-1844_1	3224	

2F	CAAAAATAACTCACATTGACATCAA	BrC_37_001_40-284-1268_1	(no match)	b: 2945
2R	TCAGAACAGGAAGGTCAATTTC	BrC_37_001_40-284-1268_2	2070	

**Sequencing primers**				

3F	GGTGCTGGATTCATGCTTTAC	BrC_03_002_4-32-317-1572-1	1123	
3R	CTGATTGCAGGAGGACACAA	BrC_03_002_4-32-317-1572-2	(no match)	
4F	GGGCATGCCATCTGTTATATG	BrC_03_001_3-13-638-284-2	(no match)	

## Competing interests

The authors declare that they have no competing interests.

## Authors' contributions

DJM, TC, PB, JS and DEa developed the method and scripts for data processing. DEa, CD, KL and MI designed and implemented the web server. MMcK produced the majority of the hosted short read data. AH, MMcK and JB performed the validation and analysis. DEd conceived of the study participated in its design and coordination. DEd together with JB helped to draft the manuscript. All authors read and approved the final manuscript.
